# The relationship between anthropometric indicators and health-related quality of life in a community-based adult population: A cross-sectional study in Southern China

**DOI:** 10.3389/fpubh.2022.955615

**Published:** 2022-09-28

**Authors:** Yu-Jun Fan, Yi-Jin Feng, Ya Meng, Zhen-Zhen Su, Pei-Xi Wang

**Affiliations:** ^1^School of Nursing and Health, Institute of Chronic Disease Risks Assessment, Henan University, Kaifeng, China; ^2^General Practice Center, The Seventh Affiliated Hospital, Southern Medical University, Foshan, China; ^3^School of Medical, Huanghe Science and Technology University, Zhengzhou, China

**Keywords:** health-related quality of life, obesity, anthropometric indicators, EQ-5D, community-based population

## Abstract

**Background:**

This study was designed to analyze the relationship of waist circumference (WC), body mass index (BMI), waist-to-hip ratio (WHR), waist-to-height ratio (WHtR), relative fat mass (RFM), lipid accumulation product (LAP) and health-related quality of life (HRQoL) in the community-dwelling population of southern China and to explore the independent contribution of socio-demographic characteristics, number of chronic diseases and anthropometric indicators to HRQoL in that population.

**Methods:**

This community-based cross-sectional survey studied 2,663 adults aged 18 years and older. HRQoL was assessed by the 3-level EuroQol 5-dimensional scale (EQ-5D-3L), and HRQoL were calculated using the Chinese EQ-5D-3L value set. The outcome variable was the EQ-5D-3L score (HRQoL). Cluster regression was used to analyse the independent contribution of each obesity indicator to HRQoL.

**Results:**

A total of 2,663 people participated in this study, and their mean EQ-5D-3L score was 0.938 ± 0.072. In this study, according to the results of the one-way ANOVA, HRQoL was significantly different between the groups of WHtR, WHR, RFM and LAP, respectively. The independent contributions of socio-demographic factors, number of chronic diseases and anthropometric measures to HRQoL in the whole population accounted for 76.2, 7.9, and 15.9% of the total effect, respectively.

**Conclusion:**

RFM and LAP were found to have a previously unreported negative impact on HRQoL in a community-dwelling population. In future studies, RFM and LAP could be used as new indicators of obesity to predict quality of life in humans.

## Introduction

With the development of society, people's eating behaviors and lifestyles have changed significantly, causing a significant increase in overweight and obesity, and the prevalence of these conditions is rapidly increasing not only in developed countries but also in developing countries, becoming a serious public health problem on a global scale (1). As of 2020, more than half of adult residents in China were overweight or obese, and the prevalence of overweight and obesity was 19% in young people aged 6–17 years and 10.4% in children under 6 years old (2). With the increasing number of obese people, obesity has been recognized as a public health problem in the Report on the Status of Nutrition and Chronic Diseases in China (2020) (3). Obesity is a risk factor for many chronic diseases, and numerous studies have shown that overweight and obesity increase the risk of diabetes, hypertension, coronary heart disease, and many other diseases (4, 5). It has also been shown that obesity and its comorbidities also come with a significant psychosocial burden, impacting numerous areas of psychosocial functioning (6, 7).

In addition, evidence from the Framingham Heart Study suggests that an increased risk of disease can lead to a large reduction in life expectancy (8). Many medical conditions associated with obesity not only increase the risk of death but also potentially affect the individual's health-related quality of life (HRQoL) (9). HRQoL is currently receiving increasing attention as a good, accurate indicator of health status. This variable provides a comprehensive assessment of a subject's physical activity functioning, mental health and social adjustment as well as the subject's self-perception of life and health (10, 11). A growing number of studies suggest that obesity is a risk factor for reduced HRQoL (12–14). Sach's study showed, after controlling for confounding factors, that obese people tended to have lower HRQoL than to people of normal weight (15). Jia (16) also showed that HRQoL decreased as the severity of obesity increased. Compared with normal-weight respondents, persons with severe obesity had significantly reduced scores on the 12-Item Short Form Survey Physical Component Summary (PCS-12) and Mental Component Summary (MCS-12), EuroQol 5-dimensional scale (EQ-5D), and EuroQol visual analog scale (EQ VAS). Persons who were overweight or moderately obese also had significantly poorer HRQoL than people of normal weight.

Currently, body mass index (BMI), waist circumference (WC), waist-to-height ratio (WHtR) and waist-to-hip ratio (WHR) are the most commonly used criteria for obesity. However, these traditional obesity indices reflect only the degree of overweight and abdominal obesity and do not distinguish between subcutaneous and visceral fat (17). Furthermore, unlike abdominal obesity (indicated by WC and WHtR) and general obesity (indicated by BMI), peripheral adiposity and larger hip circumference may offer protection from T2DM, cerebrovascular disease, and premature death (18). Among the population, the mechanism of the obesity paradox is largely due to better nutritional status and higher muscle retention (19). Considering the opposing effects of central obesity and peripheral adiposity, an indicator that assesses both masses simultaneously may better evaluate the risk of obesity on HRQoL than indicators that separately estimate either central obesity or peripheral adiposity; for example, waist–hip ratio (WHR). However, WHR may mask central obesity if both hip circumference and WC increase. Recent studies have used the relative fat mass (RFM) and the lipid accumulation product (LAP) index to assess the percentage of total body fat and the degree of obesity. Those studies have shown that RFM and LAP correlate significantly with cardiovascular disease and are better cardiovascular risk indicators than BMI or WC (20–22). Therefore, the obesity paradox in populations may be related to the use of BMI and WC, which are prone to measurement accuracies and mixed nutritional factors and are, thus, not suitable for evaluating obesity in populations, especially in the elders. However, no scholar has yet studied the relationship among RFM, LAP and HRQoL. Furthermore, research on the relationship between obesity and HRQoL in the community is very limited. The present study assumed that BMI, WC, WHR, WHtR, RFM, and LAP were negatively correlated with HRQoL.

Therefore, considering the increasing prevalence of obesity and the lack of detailed studies on the relationship between novel anthropometric indicators and HRQoL, we conducted a cross-sectional analysis of adults aged ≥18 years in southern China. Therefore, this study aimed to use a population-based survey to examine the association between HRQoL and different anthropometric indicators and to explore the different influences and independent effects of these indicators on the HRQoL of community residents. The findings may complement current research on the relationship between obesity and HRQoL and may also provide supporting information for healthcare professionals and policymakers to provide services and develop programs to improve the HRQoL of the Chinese population.

## Materials and methods

### Study design and sample

**Figure 1:**
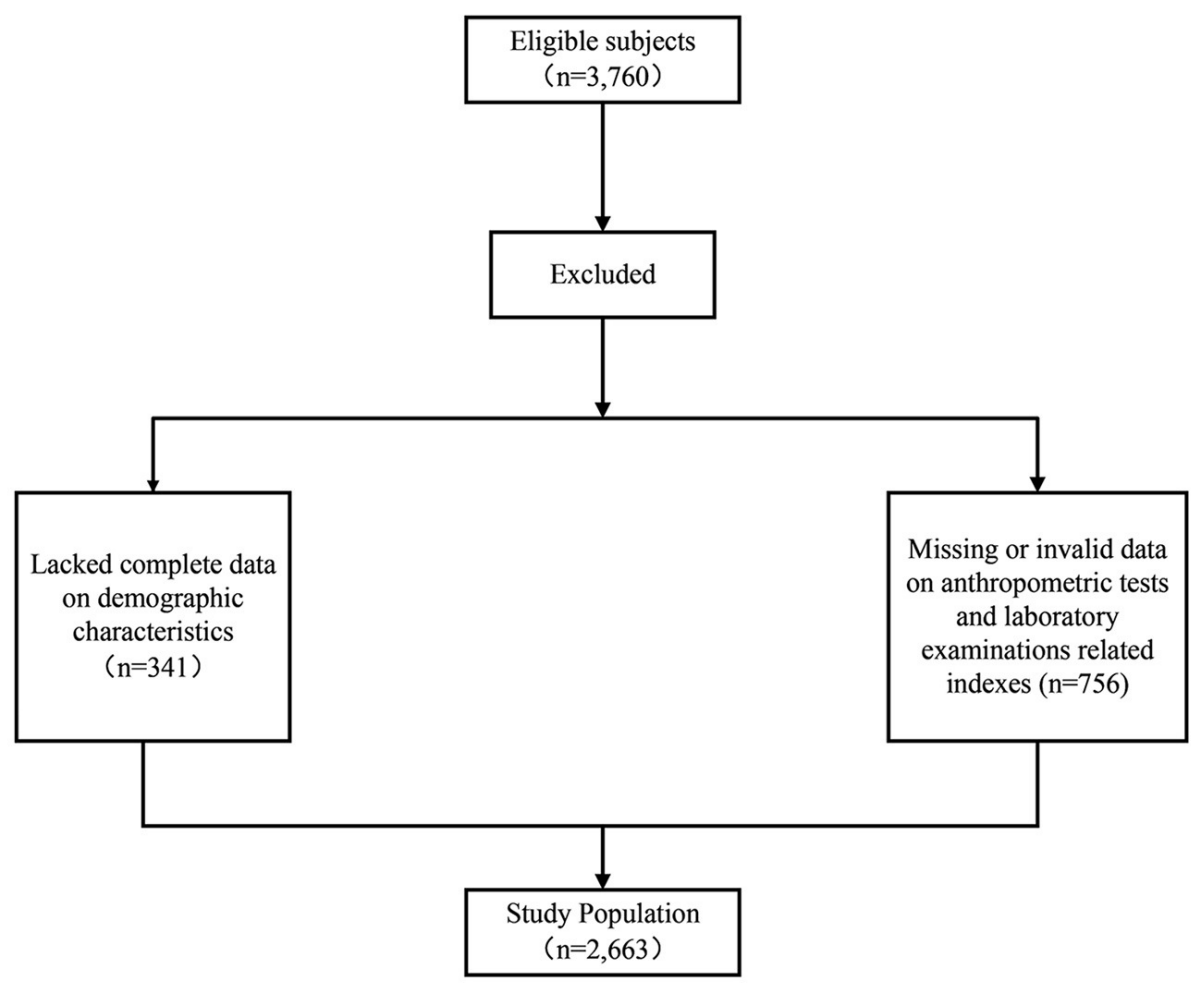
Flowchart in the selection of study population.

A cross-sectional community-based health survey was conducted in Foshan City, Guangdong Province, southern China. Participants were recruited in March 2017. The detailed sampling strategy for this survey has been described in a published study by our research team (21). Given our focus on adult demographics, we only analyzed respondents aged 18 years and older. Participants were excluded from the study if they had physical dysfunctions that may potentially affect the measurements and those who had not completed the questionnaires or had not completed the physical fitness tests. There were 3760 eligible subjects, among which 341 subjects were excluded for the lack of complete data on demographic characteristics and 756 subjects were excluded for the missing or invalid data on anthropometric tests and laboratory examinations related indexes. After excluding, a total of 2,663 adult respondents were included in this study (Figure 1).

### Data collection

With the assistance of well-trained investigators, standardized questionnaires were used to collect information about demographic characteristics (gender, age, marital status, education level, and mean monthly income), number of chronic diseases (e.g., hypertension, diabetes), and anthropometric indicators (WC, BMI, WHR, WHtR, RFM, and LAP).

#### General study questionnaire

Information on participants was gathered through a standard questionnaire including gender, age, marital status, educational level, monthly income, and chronic conditions. The chronic diseases considered in the survey included hypertension, dyslipidaemia, diabetes, stroke, cardiopathy, chronic gastroenteritis, cancer, osteoporosis, and bronchial asthma; subjects self-reported whether they suffered from each of these conditions. Marital status was classified as “unmarried,” “married,” or “other;” “divorced/widowed” was regarded as “other.” Educational level was categorized as “primary school or lower,” “junior high school” and “senior high school or above.” In this study, the definition of “unemployed' included all subjects who were out of work, regardless of whether they were looking for work or not (23).

#### HRQoL assessment tool

HRQoL was measured by the 3-level version of the EQ-5D (the EQ-5D-3L), which included both a health description system (EQ-5D index) and Visual Analog Scale (VAS). The EQ-5D-3L is a generic HRQoL measure which can compare HRQoL in populations (11, 24). This instrument has been widely demonstrated to have good reliability and validity in different populations. The first section records self-assessed health status based on five dimensions: mobility, self-care, daily activities, pain/discomfort, and anxiety/depression. Each dimension consists of three levels: no problems, some problems, and extreme problems (25). A total of 243 health state can be expressed by combining the different level from each dimension. This is then transformed into a weighted health state index score (EQ-5D index) by the Chinese time trade-off value, which is a conversion weight from health utility measurements designed based on the HRQoL preferences of Chinese populations. The Chinese form of the EQ-5D indicator has a value range of – 0.149 to 1 (26). The second component is used to assess the level of self-perceived health, ranging from 0 to 100. On this scale, 0 represents the worst conceivable state of health, and 100 represents the best conceivable state of health. The VAS can be invoked as a quantitative measure of a participant's self-judged health outcomes.

In this study, the EQ-5D health utility score was used to assess health-related quality of life. The health status was converted into a score using a utility value conversion table based on the respondents' choice of 3 levels of the 5 dimensions, with scores ranging from – 0.149 to 1 (26).

#### Anthropometric tests and laboratory examinations

Participants wore light clothing, took off their shoes, and had their weight and height measured by staff. WC (cm) was recorded at a level 1 cm above the navel. Hip circumference (cm) was measured at the level of the rearmost part of the hips, with the participant standing naturally. All the above measurements were performed twice, and the average value of the two measurements was the final measurement value. Subjects were fasted (fasted for at least 8 h) in the early morning, and blood samples were harvested from the median cubital vein by a medical professional to assess their fasting glucose, total cholesterol, and triglyceride levels. Blood collection was carried out by clinical staff and nurses following standard procedures (21).

BMI was calculated as weight in kilograms divided by the square of height in meters. The waist-to-height ratio (WHtR) was calculated as [WC (cm)/height (cm)]. The WHR was defined as the participant's WC (cm) divided by the participant's hip circumference (cm). RFM was calculated as [64 – (20× (height/WC)) + (12× sex)]. In the formula, height and WC are expressed in meters, and sex = 0 for males and 1 for females (20). LAP was calculated as (WC−60.6) × (TG [mmol/L]) in males and (WC−54.1) × (TG [mmol/L]) in females based on actual data from the population of South China (21). The cut-off points for BMI, WC, WHR, RFM, WHtR and LAP quartiles are shown in **Table 2**.

### Statistical analysis

The SPSS 24.0 were used for data analysis (Chicago, IL, USA). The association among socio-demographic, chronic diseases, anthropometric indices, and HRQoL was assessed using univariate and multivariate analyses. Univariate analyses included one-way ANOVA, and multivariate analysis was performed by entering variables in a clustered multiple linear regression analysis, in which HRQoL was used as a dependent variable, and the variables in the three clusters were used as independent variables. A two-sided statistical significance level of 0.05 was applied for all analyses.

**Figure 2:**
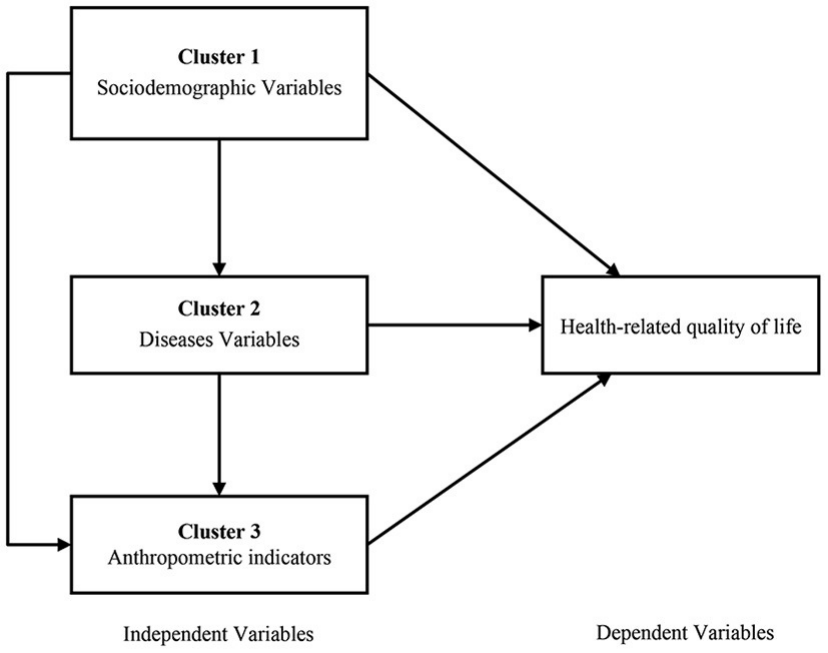
The clustered multiple linear regression model and multidirectional associations (the direction of the impact is indicated by the direction of the arrows).

Specifically, clustered multiple linear regression analysis was used to explore the effects of socio-demographic variables, number of chronic diseases, and anthropometric indicators (3 clusters based on the nature of the study variables) on the HRQoL of community residents and to estimate their independent contributions to HRQoL. The model considered the possibility of a multidirectional association between the 3 clusters of the independent and dependent variables, as shown in Figure 2. In other words, socio-demographic variables (Cluster 1) may influence the number of chronic diseases (Cluster 2), with anthropometric indicators (Cluster 3), and the dependent variable (HRQoL). Similarly, Cluster 2 may affect Cluster 3 and the dependent variable. Cluster 3 may affect only the dependent variable. Therefore, variables in the former cluster may affect variables in the latter cluster, but not vice versa. We determined the final regression model in 3 steps, which were described in a previous study (27, 28): (1) an entry regression for HRQoL for the Cluster 1 variable; (2) the equation derived in step 1 was used as a fixed part of the new regression model for the Cluster 2 variable; and (3) the equation derived in step 2 was used as a fixed part of the new regression model for the Cluster 3 variable. The inclusion and exclusion criteria for entering variables into the regression model were *P* values of 0.05 and 0.10, respectively.

The independent effect of each cluster on the dependent variable was established by calculating the corresponding R^2^ value. Then, the independent contribution of each cluster was calculated by (individual R^2^ change/total R^2^ change) × 100% (29).

## Results

### Participant characteristics

In our analyses, a total of 2,663 participants aged 18 and above were enrolled, including 1356 males (50.9%) and 1307 females (49.1%). For males, 62.09% of respondents had received only a junior high school education or less. A total of 79.13% of men are working, and more than half of the respondents are under a monthly income of less than 3,000 RMB. Only 33.21% of women had received a senior high school education or higher. Regarding marital status, 13.08% of females were unmarried, 80.87% were married, and 6.04% were divorced or widowed. Table 1 displays the demographic characteristics of the subjects.

**Table 1:** Comparison of HRQoL of community residents with different demographic characteristics by gender (*n* = 2,663).

**Variables**	**Male (*****n*** = **1,356)**		**Female (*****n*** = **1,307)**	
	***n* (%)**	**Utility value**	***n* (%)**	**Utility value**
**Cluster1:Sociodemographic factors**				
**Age groups (y)**				
18–45 (1)	794 (58.55)	0.954 ± 0.064	747 57.15)	0.945 ± 0.100
46–69 (2)	543 (40.04)	0.927 ± 0.114^*^	540 (41.32)	0.922 ± 0.118^*^
70–(3)	19 (1.40)	0.912 ± 0.117^*^	20 (1.53)	0.896 ± 0.107^*^
*P* value (F statistics)		< 0.001 (16.129)^b^		< 0.001 (8.603)^b^
**Education level**				
Primary school or lower (1)	310 (22.86)	0.916 ± 0.131	438 (33.51)	0.913 ± 0.134
Junior high school (2)	532 (39.23)	0.950 ± 0.063^*^	435 (33.28)	0.950 ± 0.053^*^
Senior high school or above (3)	514 (37.91)	0.952 ± 0.078^*^	434 (33.21)	0.940 ± 0.119^*^
*P* value (F statistics)^b^		< 0.001 (18.517) ^b^		< 0.001 (13.974)^b^
**Marital status**				
Unmarried (1)	262 (19.32)	0.946 ± 0.105	171 (13.08)	0.956 ± 0.033
Married (2)	1050 (77.43)	0.944 ± 0.074	1057 (80.87)	0.933 ± 0.115^*^
Others^a^ (3)	44 (3.24)	0.890 ± 0.215^*^^,^^†^	79 (6.04)	0.903 ± 0.121^*^^,^^†^
*P* value (F statistics)^b^		< 0.001 (7.937)^b^		0.001 (6.563)^b^
**Employment status**				
Employed (1)	1,073 (79.13)	0.956 ± 0.033	905 (69.24)	0.942 ± 0.098
Retirement (2)	167 (12.32)	0.895 ± 0.138^*^	250 (19.13)	0.902 ± 0.153^*^
Unemployed (3)	116 (8.55)	0.892 ± 0.220^*^	152 (11.63)	0.944 ± 0.064^†^
*P* value (F statistics)^b^		< 0.001 (58.048)^b^		< 0.001 (14.487)^b^
**Monthly personal income**				
< 3,000 RMB/mo (1)	695 (51.25)	0.931 ± 0.114	755 (57.77)	0.927 ± 0.120
3,000–4,999 RMB/mo (2)	479 (35.32)	0.957 ± 0.027^*^	390 (29.84)	0.942 ± 0.105^*^
5,000~6,999 RMB/mo (3)	115 (8.48)	0.947 ± 0.096	92 (7.04)	0.957 ± 0.024^*^
≥7,000 RMB/mo (4)	67 (4.94)	0.954 ± 0.046^*^	70 (5.36)	0.948 ± 0.053
*P* value (F statistics)^b^		< 0.001 (8.591)^b^		0.014 (3.547)^b^
**Cluster2 Diseases factors**				
**Number of chronic diseases**				
0 (1)	527 (38.86)	0.953 ± 0.074	601 (45.98)	0.951 ± 0.075
1 (2)	441 (32.52)	0.946 ± 0.078	403 (30.83)	0.930 ± 0.133^*^
≥2 (3)	388 (28.61)	0.926 ± 0.114^*^^,^^†^	303 (23.18)	0.908 ± 0.124^*^^,^^†^
*P* value (F statistics)^b^		< 0.001 (10.980)^b^		< 0.001 (16.352)^b^

a Others: Divorced or widowed; RMB = renminbi; (1) = reference group.

b Based on One-way ANOVA.

* Compared with (1) *P* < 0.05.

† Compared with (2) *P* < 0.05.

The score of EQ-5D-3L is shown in Table 1. The results obtained from univariate analyses indicated that HRQoL for males were between-group differences in age, education, marital status, employment status, monthly personal income, and chronic disease. And the differences were statistically significant (all *P* < 0.05). However, the HRQoL for females were between-group differences in age, education level, marital status, employment status, monthly personal income, and chronic diseases. And the differences were statistically significant (all *P* < 0.05).

### Association of HRQoL with different anthropometric indicators

Table 2 presents the HRQoL at different anthropometric indicators (BMI, WC, WHtR, WHR, RFM, and LAP) for males and females. From the results of statistical analysis, it can be observed that HRQoL decrease with increasing quartiles of anthropometric indicators (BMI, WC, WHtR, WHR, RFM, and LAP). Among the male population, the HRQoL was significantly different among quartiles of WHtR, WHR and RFM. For females, there were significant differences in HRQoL among quartiles of WC, WHtR, WHR, RFM, and LAP (all *P* < 0.05).

**Table 2:** Comparison of HRQoL of community residents with different levels of anthropometric indicators by gender (*n* = 2,663).

**Variables**	**Male (*****n*** = **1,356)**		**Female (*****n*** = **1,307)**	
	***n* (%)**	**Utility value**	**n (%)**	**Utility value**
**BMI**				
Q1: < 20.64	297 (21.90)	0.935 ± 0.119	368 (28.16)	0.941 ± 0.100
Q2:20.64-	313 (23.08)	0.953 ± 0.037^*^	345 (26.40)	0.935 ± 0.093
Q3:22.86-	368 (27.14)	0.940 ± 0.098^†^	304 (23.26)	0.934 ± 0.117
Q4:≥25.00	378 (27.88)	0.942 ± 0.083	290 (22.19)	0.926 ± 0.127
*P* value (F statistics)		0.074 (2.314)^a^		0.409 (0.964)^a^
**WC**				
Q1: < 74.00	222 (16.37)	0.949 ± 0.059	396 (30.30)	0.950 ± 0.062
Q2:74.00-	404 (29.79)	0.947 ± 0.064	392 (29.99)	0.933 ± 0.098^*^
Q3:80.97-	297 (21.90)	0.938 ± 0.120^*^^,^^†^	262 (20.05)	0.914 ± 0.175^*^^,^^†^
Q4:≥87.00	433 (31.93)	0.939 ± 0.097^*^^,^^†^	257 (19.66)	0.935 ± 0.088^‡^
*P* value (F statistics)		0.314 (1.187) ^a^		0.001 (5.834)^a^
**WHtR**				
Q1: < 0.46	399 (29.42)	0.950 ± 0.056	358 (27.39)	0.947 ± 0.070
Q2:0.46-	268 (19.76)	0.951 ± 0.048	229 (17.52)	0.947 ± 0.067
Q3:0.50-	376 (27.73)	0.935 ± 0.117^*^^,^^†^	313 (23.95)	0.915 ± 0.171^*^^,^^†^
Q4: ≥0.54	313 (23.08)	0.935 ± 0.109^*^^,^^†^	407 (31.14)	0.932 ± 0.091^‡^
*P* value (F statistics)		0.011 (3.721)^a^		< 0.001 (6.017) ^a^
**WHR**				
Q1: < 0.84	236 (17.40)	0.949 ± 0.052	400 (30.60)	0.955 ± 0.026
Q2:0.84-	255 (18.81)	0.951 ± 0.057	290 (22.19)	0.932 ± 0.120^*^
Q3:0.88-	490 (36.14)	0.947 ± 0.067	379 (29.00)	0.922 ± 0.145^*^
Q4: ≥0.92	375 (27.65)	0.927 ± 0.137^*^^,^^‡^	238 (18.21)	0.924 ± 0.109^*^
*P* value (F statistics)		0.001 (5.462)^a^		< 0.001 (7.534)^a^
**RFM**				
Q1: < 23.52	655 (48.30)	0.951 ± 0.053	9 (0.69)	0.951 ± 0.029
Q2:23.52-	548 (40.41)	0.938 ± 0.104^*^	113 (8.65)	0.955 ± 0.026
Q3:28.69-	148 (10.91)	0.927 ± 0.139^*^	506 (38.71)	0.946 ± 0.073
Q4: ≥30.01	5 (0.37)	0.870 ± 0.003^*^	679 (51.95)	0.923 ± 0.136^†^^,^^‡^
*P* value (F statistics)		0.002 (4.913)^a^		< 0.001 (5.979)^a^
**LAP**				
Q1: < 13.68	333 (24.56)	0.950 ± 0.051	327 (25.02)	0.955 ± 0.029
Q2:13.68-	314 (23.16)	0.943 ± 0.078	350 (26.78)	0.945 ± 0.088
Q3:26.52-	361 (26.62)	0.934 ± 0.120^*^	311 (23.79)	0.915 ± 0.145^*^^,^^†^
Q4: ≥43.83	348 (25.66)	0.944 ± 0.090	319 (24.41)	0.922 ± 0.134^*^^,^^†^
*P* value (F statistics)		0.136 (1.850)^a^		< 0.001 (10.049)^a^

a Based on One-way ANOVA.

BMI, body mass index; WC, waist circumference; WHtR, waist-to-height ratio; WHR, waist-to-hip ratio; RFM, relative fat mass; LAP, lipid accumulation product.

* Compared with (1) *P* < 0.05;

† Compared with (2) *P* < 0.05;

‡ Compared with (3) *P* < 0.05.

### Clustered multiple linear regression analysis

After adjustment for variables, our results showed that socio-demographics and anthropometric indicators were demonstrated to be significant predictors of the HRQoL. In the total population, the independent contributions of sociodemographic variables and anthropometric indicators to HRQoL were 76.2 and 15.9%, respectively. In the male population, the independent contribution of sociodemographic variables and anthropometric indicators to HRQoL was 86.9 and 10.3%, respectively. In the female population, the independent contributions of sociodemographic variables and anthropometric indicators to HRQoL were 55.2 and 31.0%, respectively (as shown in Tables 3, 4). On these subscales, in the overall population and in males specifically, residents with high BMI had worse HRQoL than those with low BMI. Among women, residents with greater WC had worse HRQoL than those with smaller WC.

**Table 3:** Clustered multiple linear regression analysis of the HRQoL of community residents (*n* = 2,663).

**Independent variables**	**Beta^†^**	***P* level ^a^**	**Adjusted R^2^^‡^**	**Independent contribution^§^%**
**Cluster1 (Sociodemographic factors)**				
Junior high school	0.097	< 0.001		
Senior high school or above	0.059	0.035		
Marital status (Unmarried)	0.121	0.002		
Marital status (Married)	0.106	0.005		
Employment status (Employed)	0.114	< 0.001		
Total			0.048	76.2
**Cluster2 (number of chronic diseases)**				
Number of chronic diseases (1)	– 0.045	0.033		
Number of chronic diseases (≥2)	– 0.087	< 0.001		
Total			0.053	7.9
**Cluste3 (anthropometric indicators)**				
BMI (Q2)	0.055	0.026		
BMI (Q3)	0.067	0.016		
BMI (Q4)	0.077	0.009		
WC (Q3)	– 0.087	0.003		
WHtR (Q3)	– 0.062	0.007		
WHR (Q3)	– 0.051	0.045		
WHR (Q4)	– 0.065	0.022		
RFM (Q3)	– 0.051	0.023		
RFM (Q4)	– 0.062	0.041		
LAP (Q3)	– 0.066	0.045		
LAP (Q4)	– 0.077	0.010		
Total			0.063	15.9

a *P* level: Based on Clustered multiple linear regression analysis.

† Beta is the standardized regression coefficient derived from the multiple linear regression, indicating the change in standard units of dependent variable for each increase of one standard unit in the independent variable, controlling for all other independent variables.

‡ Adjusted R^2^: is the proportion of variance in the dependent variable (Utility value) explained by the independent variables included in each regression model.

§ The independent contribution of each cluster of predictors to the HRQoL of community residents calculated as individual corresponding R^2^ change/total R^2^ change in each final model × 100%.

**Table 4:** Clustered multiple linear regression analysis of the HRQoL of male and female community residents (*n* = 2,663).

**Independent variables**	**Beta^†^**	***P* level^a^**	**Adjusted R^2^^‡^**	**Independent contribution^§^%**
**Male**				
**Cluster1 (Sociodemographic factors)**				
Junior high school	0.075	0.039		
Senior high school or above	0.080	0.043		
Marital status (Unmarried)	0.202	0.001		
Marital status (Married)	0.213	0.001		
Employment status (Employed)	0.274	< 0.001		
Total			0.093	86.9
**Cluster2 (number of chronic diseases)**				
Number of chronic diseases (≥2)	– 0.075	0.016		
Total			0.096	2.8
**Cluster3**				
BMI (Q2)	0.107	0.004		
BMI (Q3)	0.112	0.013		
BMI (Q4)	0.147	0.004		
WC (Q3)	– 0.091	0.040		
WC (Q4)	– 0.110	0.040		
WHtR (Q3)	– 0.121	0.019		
WHtR (Q4)	– 0.144	0.019		
RFM (Q2)	– 0.093	0.030		
RFM (Q3)	– 0.102	0.017		
LAP (Q4)	0.088	0.047		
Total			0.107	10.3
**Female**				
**Cluster1 (Sociodemographic factors)**				
Junior high school	0.107	0.002		
Marital status (Unmarried)	0.110	0.029		
Employment status (Retirement)	– 0.102	0.024		
Total			0.032	55.2
**Cluster2 (number of chronic diseases)**				
Number of chronic diseases (1)	– 0.076	0.011		
Number of chronic diseases (≥2)	– 0.112	< 0.001		
Total			0.040	13.8
**Cluster3 (anthropometric indicators)**				
WC (Q3)	– 0.101	0.010		
WC (Q4)	0.132	0.037		
WHtR (Q3)	– 0.080	0.017		
WHtR (Q4)	– 0.144	0.005		
WHR (Q2)	– 0.068	0.045		
WHR (Q3)	– 0.085	0.029		
WHR (Q4)	– 0.089	0.022		
LAP (Q3)	– 0.119	0.011		
LAP (Q4)	– 0.108	0.035		
Total			0.058	31.0

a *P* level: Based on Clustered multiple linear regression analysis.

† Beta is the standardized regression coefficient derived from the multiple linear regression, indicating the change in standard units of dependent variable for each increase of one standard unit in the independent variable, controlling for all other independent variables.

‡ Adjusted R^2^: is the proportion of variance in the dependent variable (Utility value) explained by the independent variables included in each regression model.

§ The independent contribution of each cluster of predictors to the HRQoL of male and female community residents calculated as individual corresponding R^2^ change/total R^2^ change in each final model × 100%.

**Figure 3:**
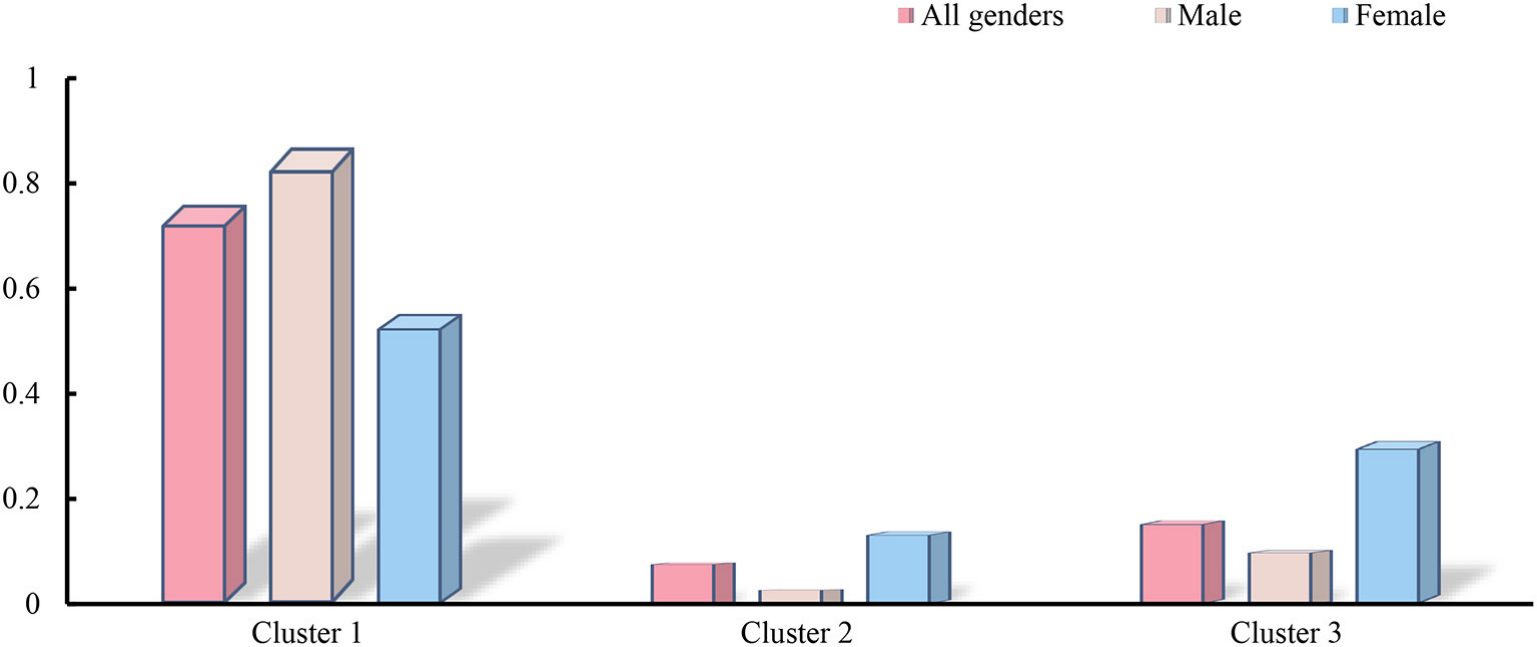
The panel shows the independent contributions of the 3 clusters to the health utility values. Cluster1 include Sociodemographic factors, Cluster2 include Diseases factors and Cluster3 include Anthropometric indicators.

The independent contributions of the 3 abovementioned clusters to HRQoL in the overall population, in males specifically, and in females specifically are illustrated in Figure 3.

## Discussion

This study investigated the relationship between anthropometric indicators and HRQoL in community residents in southern China. In this study, six anthropometric indicators, BMI, WC, WHtR, WHR, RFM, and LAP, were used to compare and assess the associations with HRQoL. It was observed in this study that WHtR, WHR, RFM, and LAP were negatively associated with HRQoL. This may be related to the fact that the RFM and LAP in the community resident groups reflect the nutritional status and functional maintenance status of the body (20). This study is the first to record the association between RFM, LAP and HRQoL.

The EQ-5D score in this sample was 0.938 ± 0.072, slightly higher than the value reported in the fifth Health Service Survey of Shanxi Province in 2013 (30). The reason for this difference may be due to the time of the survey and the different distribution areas of the sample. First, compared with 2013, the level of medical care (31), social welfare (32), and people's living standards have significantly improved. And some studies have shown that social welfare engagement can significantly improve people's quality of life (33), so the quality of life of the participants in this study will be better than the survey population in 2013. Secondly, the survey population in this study is from the south, which is more economically prosperous than the north and has a more developed health insurance system. Since residents in the southern region have better medical benefits (34). And some studies have shown that participants with better economic status have better health-related quality of life (35), so standard of living and HRQoL in this study might be higher. We found that the differences in age, sex, education, employment and marital status of HRQoL in this study were consistent with the published literature (36–38). As people age, they can develop a range of health problems that can affect their HRQoL (39). At the same time, older participants tended to have more physical and cognitive functioning problems than younger participants (40), possibly leading to a decrease in HRQoL with age. In addition, the results of this study showed that the decline in quality of life may be influenced by the state of work. Retired and unemployed people have worse HRQoL than those who are employed. This may be due to the unstable mental state of the unemployed person Some studies have shown that the unemployed—the long-term unemployed in particular—exhibit higher levels of distress, psychiatric symptoms, and self-harm than the employed (23, 41, 42). Those who have just retired are not yet comfortable with the change in role and may also be affected by aspects of their physical condition (43, 44). This is consistent with a Korean study on the quality of life after retirement for different age groups (45). The most significant factors affecting quality of life for adults in midlife were financial status and mental health, while for those in their 50s, mental health and family relationships were the primary factors, followed by physical states (46). The HRQoL observed in our study was generally higher in males than in females, consistent with previous findings (47–49). However, inconsistent with another study on the EQ-5D-5L criteria for the Chinese urban population, their findings showed that women had greater HRQoL than men, possibly due to the different composition of the two study samples and the fact that women were in a higher socio-economic group in their study (50).

There is limited research on the effect of obesity on HRQoL in community populations, with most studies focusing on the relationship between BMI and HRQoL. Studies of this effect in Spanish adults have shown that HRQoL decreases with decreasing BMI (51). Most studies in adults have assessed HRQoL using the 36-Item Short Form Survey (SF-36) or the EQ-5D, confirming a significant negative correlation between BMI and HRQoL (7, 13, 14). In contrast, the obesity paradox in the elderly population shows that obese elderly and obese patients have higher HRQoL, better prognosis, lower disability and lower mortality than normal weight elderly and chronically ill patients (19, 52, 53). Nonetheless, no statistically significant association was found between BMI and HRQoL in this study. However, the criteria used to assess obesity in these studies may make it difficult to accurately measure height in older adults due to the natural progression of aging. Second, it is difficult to exclude the effects of nutrition and muscle retention from WC measurements; therefore, the conclusion that obesity facilitates the maintenance of a better HRQoL is biased. Furthermore, WHR is often used to balance the relationship between fat distribution and nutrition or muscle retention; however, this measurement may mask central obesity if both hip circumference and WC increase (54, 55). In our study, we found that women's HRQoL was more sensitive to WC than men's HRQoL. This may be because women may be more vulnerable to weight and body image than men (56). Excessive dieting to keep fit may lead to reduced HRQoL in women. This can also be explained by cultural beliefs about one's weight and the increased discrimination against overweight women in work-related life and social roles (57). In this study, we introduced new obesity assessment criteria, such as RFM and LAP, and the analysis found that increases in WHtR, WHR, RFM, and LAP all led to a decrease in HRQoL. This may be because LAP, a new obesity index based on WC and triglycerides (TGs), is a useful indicator of visceral fat (58). RFM, which is based on the ratio of height to WC, is a more accurate estimate of body fat percentage than BMI. Furthermore, some studies have shown that RFM and LAP are significantly associated with cardiovascular disease. As RFM and LAP increase, the probability of people developing cardiovascular disease also increases (20–22, 59–61). Therefore, this may lead to a reduction in people's HRQoL.

Our study found that the independent contribution of the first cluster (socio-demographic characteristics) to HRQoL was much greater than that of the other two clusters, not only in the whole population but also in males and females separately. This may be because the EQ-5D-3L appears to be more sensitive in distinguishing between socio-demographic subgroups based on age, gender, marriage, education, employment and monthly income (36). We also found that in these populations, the independent contribution of the anthropometric indicators to HRQoL was even greater than the chronic disease prevalence. This may be partly because in most of these chronic disease categories, obesity is likely to be the cause of the disease (62).

## Limitations

This study has several limitations that should be noted. First, the data on socio-demographic variables and chronic diseases in our research were obtained from self-reports, which might lead to biases or inaccuracies. This study did not consider the severity of chronic disease, which may have an effect on HRQoL. Second, some possible risk factors were not collected, including exercise, smoking, and drinking. Future studies will need to provide more detailed information. In addition, this study may have overestimated some parameters due to the ceiling effect of EQ-5D-3L. Third, this study used cross-sectional survey data to analyse the independent contribution of these three clusters to HRQoL, so the observed results cannot be supposed to be causal. Further in-depth studies of longitudinal follow-up data are needed to explore the causal relationship between them.

## Conclusion

Despite its limitations, this study analyzed the correlation between obesity-related indicators of obesity (specifically RFM and LAP) and HRQoL in a community-based population. In this study, HRQoL decreased as BMI, WC, WHtR, WHR, RFM and LAP increased, and these results suggest that the accumulation of fat has a negative impact on HRQoL. In future measurements, RFM and LAP could be used as measures of nutrition and obesity. Additionally, our findings suggest that HRQoL is mainly influenced by marital status, education level and work status. When developing future interventions, the relevant authorities should pay increased attention to people with low educational attainment as well as people who are unmarried, divorced, widowed, unemployed, or retired.

## Data availability statement

The datasets supporting the findings of this article cannot be shared publicly due to privacy reasons. Requests to access the data can be directed to the corresponding author(s).

## Ethics statement

The studies involving human participants were reviewed and approved by Ethics Committee of Guangzhou Medical University. The patients/participants provided their written informed consent to participate in this study. Written informed consent was obtained from the individual(s) for the publication of any potentially identifiable images or data included in this article.

## Author contributions

Y-JFan and P-XW designed the study, analyzed the data and wrote the draft manuscript. Y-JFeng performed data analysis and wrote the draft manuscript. YM and Z-ZS collected the data and performed data analysis and edited the manuscript. P-XW finalized the manuscript with inputs from all authors. All authors contributed to the development of the study framework, interpretation of the results, revisions of successive drafts of the manuscript and approved the version submitted for publication.

## Conflict of interest

The authors declare that the research was conducted in the absence of any commercial or financial relationships that could be construed as a potential conflict of interest.

## Publisher's note

All claims expressed in this article are solely those of the authors and do not necessarily represent those of their affiliated organizations, or those of the publisher, the editors and the reviewers. Any product that may be evaluated in this article, or claim that may be made by its manufacturer, is not guaranteed or endorsed by the publisher.

## Abbreviations

HRQoL: Health-related quality of life
VAS: Visual Analog Scale
BMI: Body mass index
WC: Waist circumference
WHtR: Waist-to-height ratio
WHR: Waist-to-hip ratio
RFM: Relative fat mass
LAP: lipid accumulation product
PCS-12: 12-Item Short Form Survey Physical Component Summary
MCS-12: 12-Item Short Form Survey Mental Component Summary.
